# Telemedicine and Virtual Interventions in Cancer Rehabilitation: Practical Application, Complications and Future Potentials

**DOI:** 10.1007/s11912-024-01612-8

**Published:** 2024-11-06

**Authors:** Philip Chang, Jessica Engle

**Affiliations:** 1 250 N. Robertson Blvd, Suite 403, Beverly Hills, CA 90211 USA; 2 600 N. Wolfe Street, Meyer Building 1-163, Baltimore, MD 21287 USA

**Keywords:** Telemedicine, Telehealth, Telerehabilitation, Cancer rehabilitation, Video visits

## Abstract

**Purpose of Review:**

The purpose of this review is to provide an overview of the practical applications of comprehensive cancer rehabilitation services through telemedicine.

**Recent Findings:**

Telemedicine has been shown to be an effective platform leading to positive outcomes and high patient/provider satisfaction for several forms of skilled therapy and cancer physiatry visits. Several survivorship resources are also available through telemedicine in recent years.

**Summary:**

Telemedicine can increase accessibility to geographically sequestered services including cancer physiatry, skilled therapy and survivorship resources. In certain situations and for specific services, telemedicine can be effective, however, in other situations such as the evaluation of new neurologic deficits or when providing manual therapies, in-person visits should take precedence.

## Introduction

Since the pandemic, several clinicians have adopted a new medical delivery system in which telemedicine is now seamlessly integrated into their practice. The advantages of telemedicine are plentiful and start with convenience and ease of access. Patients being treated for active cancer already experience high levels of appointment burden and removing the commute and travel costs can significantly alleviate the process. Additionally, although society has adapted to a new normal of living with COVID, several immunocompromised patients with cancer are rightly concerned with sharing waiting rooms with others who are potentially infectious. Telemedicine allows concerned patients to receive health services while remaining quarantined. Benefits are not limited to patients as providers have also experienced enhanced workflows. Logistical and scheduling complications with limited clinic rooms are decompressed with virtual visits. Providers who work at multiple geographically dispersed sites are able to maintain care continuity as distance is no longer a factor. Patient family members who can provide additional history and insight can be added to visits at the click of a button and the potential for virtual multidisciplinary clinics with videoconferencing is also realized.

Although there are specific situations in which telemedicine is not appropriate and an in-person consultation is required, the benefits thus far appear to heavily outweigh the costs. Furthermore, as congress continues to extend the allowance for telemedicine visits, it becomes more likely that it will stay a permanent part of healthcare. We write this review to discuss the practical applications of telemedicine in cancer rehabilitation medicine, skilled therapies and wellness/resilience/survivorship.

## Practical Applications

### Pitfalls

While telemedicine services have proven advantages to patients and providers, it is not a perfect substitute for traditional in-person care. Prior to discussing telemedicine’s applications in rehabilitation, we briefly layout common pitfalls that may occur.

The process of telemedicine is not seamless and issues at times may arise. Most patients and providers have experienced the frustrations of a dropped call, poor signal reception or the inability to get audio or video working. While some of these complications can be troubleshooted and are considered a relative annoyance, other barriers may pose greater challenges including the development of new social inequities, issues surrounding privacy/ethics, changing policies/laws, and reimbursement considerations. Some barriers may be overcome with time such as lowering costs of telemedicine technologies (computers, smart phones, tablets). Others may be overcome with education and involving cargiving persons to facilitate technology when there are literacy issues [1]. Protocols will continue to evolve regarding video visits and several are starting to include the provider determining and documenting the patient’s current physical location and a contact number in the event of a medical emergency. They are also starting to include that providers document their own location as licensing laws (in addition to insurance regulations) may or may not allow patients to be seen when out of state. As these laws continue to change and previous emergency pandemic rulings continue to lapse, additional complications regarding reimbursement and scope of geographic practice will come up. Furthermore, it is possible to miss “red flag” exam findings that may have been noted at an in person visit creating potential quality of care issues [2]. Rehabilitation providers frequently screen for neurologic deficits when patients are in-person and can also assess for serious etiologies of acute pain such as malignant fractures. It is unclear to what degree such red flags are currently missed using telemedicine and should be a topic of further study. Still, the benefits of video visits are self-evident and have readily been applied to cancer rehabilitation medicine.

### Telemedicine in Cancer Rehabilitation Medicine

Cancer rehabilitation medicine is an emerging subspecialty within physical medicine and rehabilitation. It has been previously defined as, “the identification, evaluation and rehabilitation of neuromuscular, musculoskeletal, and function disorders associated with cancer and it’s treatment emphasizing the restoring and maintenance of function and quality of life.” [3] Cancer rehabilitation medicine providers (also termed cancer physiatrists) have completed residencies in physical medicine and rehabilitation and in recent years have received specialized training in a one-year cancer rehabilitation fellowship. During this time they learn how to evaluate and manage numerous cancer specific disorders including neoplasm related fatigue, trismus, comprehensive care for metastatic spinal disease, chemotherapy induced neuropathy, aromatase inhibitor arthralgias, cancer related cognitive impairment, radiation fibrosis syndrome, post-mastectomy pain syndrome, survivorship issues, neurologic complications secondary to checkpoint inhibitors, musculoskeletal graft vs. host disease and several others. While specific skills vary from provider to provider, cancer rehabilitation physicians utilize a variety of tools to address patient needs including prescription of orthotics/prosthetics, home exercise programs, pharmacologic therapy, integrative therapies, and therapeutic interventions including trigger point injections, botulinum toxin, nerve blocks, and joint/bursa injections.

As the subspeciality is relatively new and growing, there remains a dearth of providers and many still are concentrated within cancer centers in urban areas [4]. As such, telemedicine has become a vital platform which allows patients to receive this specialized care. Fortunately, several cancer physiatrists have implemented telemedicine into their practice and are able to use it to effectively provide services. A survey study among cancer physiatrists demonstrated that respondents felt telemedicine is an adequate platform for patient follow up with stable problems, providing education/counseling, and for the prescription and titration of medications [5]. The same study however reported that less than 20% of respondents reported conducting a virtual comprehensive physical exam which likely contributes to the preference for in-person evaluations for patients with new/worsening problems, evaluating for procedures, and evaluating for prosthetics/orthotics^5^. In an examination of perceived efficacy, another study evaluated patient and provider satisfaction with telemedicine services in an outpatient cancer rehabilitation clinic. They found that in the vast majority of encounters both patients and providers were satisfied with the visit and that it was effective in addressing the main problem for which patients presented [6]. Of note, there was a trend among providers for decreased satisfaction when patients were being seen for their initial encounter.

On a practical note, when seeking the services of a cancer physiatrist it is most effective to refer for in-person initial evaluations when possible. However, in situations where patients are unable to present in-person due to geographic or other constraints then video visits may be considered, particularly for specific indications. These may include evaluation/management of cancer related fatigue, lifestyle medicine counseling, cancer related cognitive impairment and preoperative counseling such as for sarcoma [2]. On the other hand, when patients require evaluation for new or worsening musculoskeletal and neurologic complaints, in-person evaluation is preferred [2].

### Telemedicine in Skilled Therapies

There are numerous types of skilled therapies available that can effectively treat a variety of conditions affecting patients with cancer. In this section we will briefly discuss the utility of each type of therapy and evidence regarding its use of telemedicine.

#### Physical Therapy

Physical therapy services focus on the examination, diagnosis and treatment of movement dysfunction. Physical therapists have a wide scope of practice for cancer survivors including physical conditioning, musculoskeletal pain issues, neoplasm related fatigue, and correction of functional impairments. Common reasons for referral to physical therapy may include deconditioning following treatment for cancer, difficulty walking secondary to chemotherapy induced peripheral neuropathy, frozen shoulder following treatment for breast cancer, strengthening exercises for someone on androgen deprivation therapy, or decreased range of motion secondary to radiation fibrosis. Physical therapists are widely available across the spectrum of healthcare services including in-patient, subacute, outpatient, and home settings. Given their relative abundance, the importance of telemedicine is less pronounced as their in-person services will more often be available. That said, several studies have demonstrated the efficacy of physical therapy services when applied through telemedicine. A longitudinal observational study in 126 patients with chronic low back pain found that participant outcomes using telehealth physical therapy was similar to that of in-person physical therapy [7]. A study on multiple systematic reviews found that telemedicine physical therapy was found to be comparable to or superior to no therapy for multiple conditions including osteoarthritis, joint replacement, multiple sclerosis and for cardiac and pulmonary rehabilitation [8].

#### Occupational Therapy

Occupational therapy interventions focus on everyday life activities including activities of daily living and instrumental activities of daily living to promote health, wellbeing and participation in occupations [9]. Additionally, occupational therapists may undergo further specialized training to become certified hand therapists focusing on hand impairments. Common reasons for referral to occupational therapy may include home evaluation (to optimize the home environment for mobility), impairment with activities of daily living such as bathing or getting dressed, hand arthralgias caused by aromatase inhibitor therapy or fine motor impairments caused by chemotherapy induced peripheral neuropathy. In contrast to physical therapists, occupational therapists are not as readily available and certified hand therapists even less so. As such, telemedicine may play a more important role here particularly for patients far from larger medical systems. A small study with 26 participants with breast cancer showed that telemedicine visits were feasible, effective and accepted by patients in the perioperative setting [10]. Hand therapy services offered through telemedicine were also found to be satisfying to patients and providers with high levels of agreement for objective clinical measures like range of motion and strength when compared with traditional clinical models [11].

#### Speech Language Pathology

Speech language pathology works to prevent, assess, diagnose, and treat speech, language, communication, cognitive impairments and swallowing disorders [12]. In oncology populations they are often utilized for patients with cancers affecting the head/neck regions, those with primary or secondary brain tumors causing cognitive/dysphagia/communication deficits, and those with cancer related cognitive impairment or “chemobrain”. While the number of speech language pathologists in the United States is comparable to occupational therapists, a significant proportion of them work in primary and secondary education and are largely unavailable to the adult cancer population [13]. Given their relative scarcity but also the fact that many of their interventions do not require hands on modalities, speech therapy may be the best natural fit for telemedicine. Multiple studies have shown good satisfaction with telemedicine visits and one study demonstrated that the majority of patients actually preferred speech telemedicine visits over in-person visits [14, 15].

#### Lymphedema Therapy

Lymphedema therapy is a specialized branch of either physical therapy, occupational therapy, or speech language pathology (head/neck lymphedema). Lymphedema therapists are trained in providing complete decongestive therapy which utilizes elements of compression, therapeutic exercise, skin care and manual lymphatic drainage (also called lymphatic massage) to reduce areas of swelling in the body that are caused by lymphedema. Some therapists may also use these interventions to reduce swelling that is not caused by lymphedema. Lymphedema therapists tend to be fewer and further in-between than traditional physical and occupational therapists and certified specialists can be found at the website www.clt-lana.org among other websites. To date, there are limited studies regarding the use of telemedicine in lymphedema therapy however as an intervention that requires hands on measurements and techniques, it seems that it would be unlikely to translate well. One study demonstrated that patients initially seen through telemedicine for lymphedema required further re-assessment in-person for objective limb size measurements and learning self-massage indicating that telemedicine may not be the best platform [16].

#### Pelvic Therapy

Pelvic therapy is another specialized form of physical therapy that focuses on pain and dysfunction of the bowel, bladder and uterus. These problems can include sexual dysfunction, coccydynia, and urinary incontinence. Interventions may include biofeedback, manual therapy, electrical stimulation and therapeutic exercise such as strengthening of the pelvic floor muscles. Similar to most other types of therapy, pelvic therapy has also been shown to be effective when delivered through telemedicine. One study examined pelvic floor telerehabilitation on older women with urinary incontinence and 71.9% of participants were completely satisfied with the program’s effects on their symptoms [17].

### Telemedicine in Wellness, Resilience and Survivorship

Patients seek comprehensive care with services for those currently in active cancer treatment, prevention of new cancers and recurrences, as well as post-treatment survivors to improve various aspects of health including physical and psychosocial well-being. Cancer rehabilitation is a key component of wellness focusing on multidisciplinary care to improve social, mental, physical, and vocational function [18]. Many patients seek to prevent loss of muscle mass and improve strength with exercise and diet. However, for those with low exercise tolerance, bone lesions, or pre-existing musculoskeletal or neurological impairments, pursuing an exercise program may be difficult. A referral to cancer rehabilitation medicine may be beneficial prior to starting an exercise program to discuss general guidelines, assess risk and set precautions, much of which can be done virtually. While a critical element of patient wellness, rehabilitation services are only a single piece of the comprehensive telemedicine services available to promote cancer survivorship.

Nutrition consults can be done virtually while a patient sits in their own kitchen browsing their existing ingredients. Healthy dietary habits are an important part of wellbeing and allows patients to take greater control of their health goals. Nutrition classes are often offered from cancer centers and community organizations and may commonly be held virtually. Group exercise classes targeted for patients with cancer that were once in-person have adopted virtual platforms which has led to increased capacity and turnout. Some patients may appreciate the “no pressure atmosphere” of virtual exercise classes that allow them to exercise in private or not be visible to the group and a chance to start something new. Some centers offer free virtual cancer support programs spanning the continuum of survivorship including aspects of physical, emotional, psychological, and spiritual health. Such classes may include reiki, yoga (chair or mat classes), meditation, monthly support groups, art therapy, survivorship education, and workshops to anyone with cancer, however, other institutions/organizations may only offer classes to specific groups of people such as those in active treatment at their facility. Various resources can be offered to patients through organizations including YMCA, Silver Sneakers, and LiveStrong [2].

In the presence of physical and mental stressors, resiliency is the ability of a person to restore or maintain physical or psychological function that is relatively stable. This concept is dynamic and may change over the course of one’s life [19]. Patients may engage in telemedicine prehabilitation to improve resiliency trying to optimize quality of life, function, nutrition, physical and mental health prior to a planned procedure or treatment, ideally close to the onset of diagnosis. Poorer survival outcomes have been associated with limitations in function prior to cancer and as a consequence of cancer and cancer-related treatment [20].

## Future Directions

Additional studies will be needed to show durability, practicality, financial costs, and outcomes with telemedicine in cancer rehabilitation. However, the future is clear and is heading towards customized exercise programming at all stages of the cancer journey from diagnosis to survivorship. Prehabilitation will likely continue to grow as a movement and telemedicine may allow for a more geographically equitable distribution. Survivorship programming whether for targeting existential distress or cancer related cognitive impairment may move to on-demand video if efficacy is on par with in-person programs. Of equal interest is the prospect of conducting research remotely and exploring which physical functional outcome measures may be validated when assessed through telemedicine.

## Conclusion

Telemedicine has repeatedly demonstrated itself to be a reliable platform for delivering care that is as effective as traditional in-person care across specialties. The longer telemedicine remains in use, the more ingrained it will become in our healthcare system and the higher the likelihood of its permanence. As such, we envision that it will be here to stay and should continue to shape our practice models assuming its presence. Numerous studies have already demonstrated that comprehensive rehabilitation care including numerous forms of skilled therapy (with the exception of a few forms of manual therapy) and physiatry care can effectively be provided via telemedicine. Moreover, it has allowed lesser available specialties to be accessible to larger patient populations.

## Key References

6. Chang PJ, Jay GM, Kalpakjian C, Andrews C, Smith S. Patient and Provider-Reported Satisfaction of Cancer Rehabilitation Telemedicine Visits During the COVID-19 Pandemic. *PM R*. Published online January 17, 2021. 10.1002/pmrj.12552.

• This study demonstrated good patient/provider satisfaction with telemedicine visits in an outpatient cancer physiatry practice.

14. Tenforde AS, Borgstrom H, Polich G, et al. Outpatient Physical, Occupational, and Speech Therapy Synchronous Telemedicine: A Survey Study of Patient Satisfaction with Virtual Visits During the COVID-19 Pandemic. *Am J Phys Med Rehabil*. 2020;99(11):977–981. 10.1097/PHM.0000000000001571.

• This study has demonstrated good patient satisfaction with virtual visits for the major skilled therapies.

16. Lopez CJ, Edwards B, Langelier DM, Chang EK, Chafranskaia A, Jones JM. Delivering Virtual Cancer Rehabilitation Programming During the First 90 Days of the COVID-19 Pandemic: A Multimethod Study. *Arch Phys Med Rehabil*. 2021;102(7):1283–1293. 10.1016/j.apmr.2021.02.002.

• This study demonstrated that for certain conditions such as lymphedema, further in-person evaluations were required as the initial telemedicine visit was inadequate.

## Data Availability

No datasets were generated or analysed during the current study.
